# Long-Term Outcome of Kidney Transplant Patients from Rural Farming Areas with Balkan Nephropathy—A Single-Centre Report

**DOI:** 10.3390/jcm15072558

**Published:** 2026-03-27

**Authors:** Luka Rukavina, Stela Živčić-Ćosić, Dean Markić, Christophe Štemberger, Jelena Ljubić, Tomislav Rukavina, Josip Španjol, Ivan Brzić, Josip Samardžić, Marija Domislović, Nikolina Bašić-Jukić, Bojan Jelaković

**Affiliations:** 1 Department of Urology, Clinical Hospital Center Rijeka, 51000 Rijeka, Croatia; lrukavina91@gmail.com (L.R.); dean.markic@medri.uniri.hr (D.M.); josip.spanjol@medri.uniri.hr (J.Š.); 2 Department of Urology, General Hospital Varaždin, 42000 Varaždin, Croatia; 3 Department of Nephrology, Dialysis and Kidney Transplantation, Department of Internal Medicine, Clinical Hospital Center Rijeka, 51000 Rijeka, Croatia; 4 Department of Internal Medicine, Faculty of Medicine, University of Rijeka, 51000 Rijeka, Croatia; 5 Department of Urology, Faculty of Medicine, University of Rijeka, 51000 Rijeka, Croatia; 6 Department of General Pathology and Pathological Anatomy, Faculty of Medicine, University of Rijeka, 51000 Rijeka, Croatia; drstembo@yahoo.com; 7 Department of Pathology and Cytology, Clinical Hospital Center Rijeka, 51000 Rijeka, Croatia; 8 Department of Infectious Diseases, County Hospital Čakovec, 40000 Čakovec, Croatia; jelljub@gmail.com; 9 Department of Social Medicine and Epidemiology, Faculty of Medicine, University of Rijeka, 51000 Rijeka, Croatia; tomislav.rukavina@medri.uniri.hr; 10 Department of Clinical Microbiology, Teaching Institute of Public Health of Primorje-Gorski Kotar County, 51000 Rijeka, Croatia; 11 Municipality of Bebrina, 35254 Bebrina, Croatia; ivanbrzic@gmail.com; 12 Surgical Services, Department of Abdominal and Thoracic Surgery, General Hospital “Dr. Josip Benčević”, 35000 Slavonski Brod, Croatia; josip.samardzic@gmail.com; 13 Faculty of Dental Medicine and Health, University of Osijek, 3100 Osijek, Croatia; 14 Department of Nephrology, Arterial Hypertension, Dialysis and Transplantation, Clinical Hospital Center Zagreb, 10000 Zagreb, Croatia; domislovic.marija@gmail.com (M.D.); nina_basic@net.hr (N.B.-J.); 15 Department of Internal Medicine, School of Medicine, University of Zagreb, 10000 Zagreb, Croatia

**Keywords:** aristolochic acid, Balkan nephropathy, kidney transplantation, long-term outcomes, nephroureterectomy, urothelial carcinoma, upper urinary tract urothelial carcinoma

## Abstract

**Background:** Balkan nephropathy (BEN) is an environmental form of aristolochic acid nephropathy (AAN) strongly associated with upper urinary tract urothelial carcinoma (UTUC). Clinical diagnosis remains challenging, and misclassification is frequent. The study reassessed BEN diagnoses in kidney transplant recipients from rural farming areas who did not undergo prophylactic bilateral nephroureterectomy and evaluated post-transplant outcomes. **Methods:** In this retrospective single-centre study, we analysed 12 kidney transplant recipients from rural Balkan farming regions. BEN diagnoses were reevaluated according to international consensus criteria. Key endpoints included patient and graft survival, post-transplant clinical course, and UTUC characteristics. **Results:** Upon reassessment, three of the six patients initially diagnosed with BEN were reclassified as having BEN. Among the remaining six patients, four were reclassified as having sporadic BEN. Overall, only 33% of patients had a diagnosis concordant with their admission records. During a median follow-up of 6.8 years (IQR 2.1–15.8), UTUC developed in seven out of 12 patients. The UTUC cases were predominantly high-grade and multifocal, and they were identified as the leading cause of death. Five-year patient and graft survival rates were 71% and 100%, respectively. **Conclusions:** BEN is frequently misdiagnosed or misclassified in kidney transplant candidates from rural farming areas. Despite excellent graft survival, the high incidence of post-transplant urothelial carcinoma underscores the necessity of accurate diagnosis and the consideration of prophylactic bilateral nephroureterectomy. Lifelong intensive surveillance is essential not only for patients from established BEN regions but also for individuals from other rural farming areas at risk for sporadic BEN.

## 1. Introduction

Balkan nephropathy (BEN) is an environmental form of aristolochic acid nephropathy (AAN) [1,2,3]. This condition is a chronic tubulointerstitial kidney disease frequently associated with upper urinary tract urothelial carcinoma (UTUC). It is predominantly observed in rural Balkan regions characterized by household clustering and prolonged residency [1]. BEN develops in individuals who have consumed bread made from wheat contaminated with seeds of Aristolochia clematitis [2]. This plant contains aristolochic acid (AA), a potent nephrotoxin, carcinogen, and mutagen [1,3,4,5,6,7,8].

Nikolić et al. proposed that BEN may occur outside traditionally recognized high-prevalence BEN regions, emerging sporadically in non-endemic agricultural villages [9]. This observation aligns with the widespread distribution of Aristolochia clematitis [7]. They identified “sporadic BEN” in patients from various Serbian regions undergoing surgery for UTUC after excluding other known nephropathies. In the absence of molecular confirmation, a diagnosis of sporadic BEN should be interpreted as a strongly suggestive clinical hypothesis. Our identification of aristolactam DNA adducts in UTUC patients from non-endemic areas of Croatia and Bosnia provided the first molecular evidence and confirmation of the existence of sporadic BEN [10].

While the detection of AA-DNA adducts or the fingerprint Tp53 gene mutation is diagnostic, molecular confirmation is not widely available. In the absence of routine biomarkers or pathognomonic signs, BEN is diagnosed based on epidemiological, clinical, and laboratory data [11]. Consequently, BEN often remains the most probable diagnosis, even if it cannot be definitively confirmed without molecular-biochemical evidence [12]. In cases of sporadic BEN, the simultaneous occurrence of UTUC, in the absence of other nephropathies, increases diagnostic accuracy [9].

Established diagnostic criteria are not always consistently adopted in routine clinical practice. A significant gap exists between expert recommendations and clinical application, which can lead to the overestimation of BEN. This discrepancy may influence critical clinical decisions, such as those concerning prophylactic bilateral nephroureterectomy prior to kidney transplantation. Conversely, sporadic BEN is often underdiagnosed. Many nephrologists may be unaware of this variant and continue to consider residency in endemic regions a *conditio sine qua non* for diagnosis, which can result in inadequate follow-up. As with other cases of end-stage kidney disease (ESKD), transplantation remains a primary renal-replacement therapy for BEN patients. However, several specific clinical considerations are noted in the literature: all BEN patients should undergo bilateral nephroureterectomy before transplantation; those who decline the procedure require close monitoring for post-transplant UTUC; and a potential reduction in immunosuppression intensity, along with conversion to mTOR inhibitors, should be evaluated by the clinical team [11].

The aim of this study was to perform a descriptive diagnostic re-evaluation of BEN within a cohort of kidney transplant recipients from rural Balkan farming regions who had not undergone prophylactic bilateral nephroureterectomy. Additionally, we analysed the post-transplant clinical course and outcomes, specifically focusing on the characteristics of *de novo* UTUC and bladder UC.

## 2. Materials and Methods

### 2.1. Study Population

**Figure 1:**
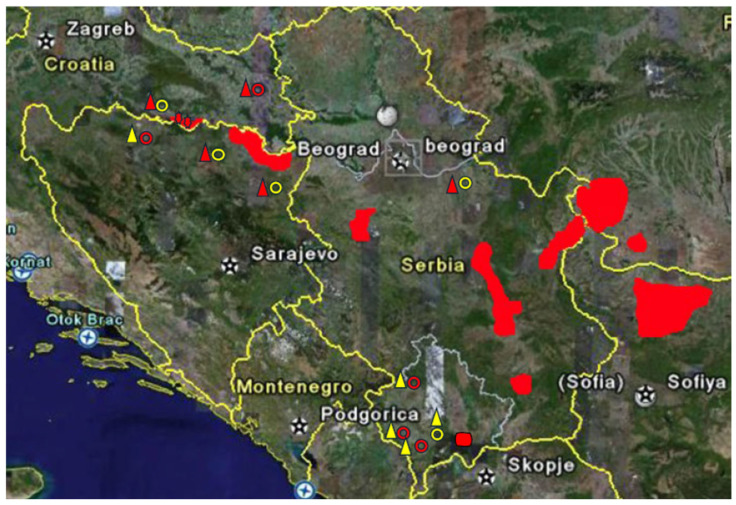
Geographic distribution of Balkan nephropathy. Farming villages where kidney transplant patients lived: red triangles—BEN diagnosed at admission; yellow triangles—non-BEN at admission; open red circles—BEN diagnosis (sporadic) after post hoc re-evaluation; open yellow circles—non-BEN diagnosis after post hoc re-evaluation; red areas represent endemic regions in different countries.

Between 1 January 1985 and 31 December 2024, a total of 969 kidney transplantations (92.4% first transplants) were performed at our centre. Of these, 82.4% (*n* = 798) were from deceased donors and 17.6% (*n* = 171) were from living donors (170 blood relatives and one spouse). Among 895 transplant recipients, 1.3% (*n* = 12) originated from rural regions of Bosnia and Herzegovina, Croatia, Kosovo and Serbia, and were referred with a diagnosis of either BEN (*n* = 6) or CKD of unknown etiology (*n* = 6) (Figure 1). All transplant-related data, including primary CKD etiology and pre- and post-transplant malignancies, were recorded in our database and reported to the Collaborative Transplant Study (CTS, www.ctstransplant.org). This retrospective, single-centre study included these 12 adult kidney transplant recipients (nine male, three female), representing all eligible cases. All patients in this cohort received a kidney transplant from a deceased donor, and none underwent prophylactic bilateral nephroureterectomy. Demographic, epidemiological, clinical, and pathological characteristics were collected from medical records, including pre-transplant evaluations, transplant-related data, and long-term follow-up on cardiovascular complications, infections, malignancies, rejection episodes, graft function, and patient and graft survival. Blood pressure and laboratory values were obtained upon hospital admission for transplantation. Anaemia was defined according to gender-specific reference intervals for the Croatian population and was managed with blood transfusions and, since the 1990s, with erythropoietin.

During the study period, the standard immunosuppressive regimen at our centre consisted of an induction agent combined with triple maintenance therapy. Exceptions included the first three transplants (1985–1986), performed prior to the availability of calcineurin inhibitors. Since the 1990s, cyclosporine and azathioprine have largely been replaced by tacrolimus and mycophenolate, and antilymphocyte sera were superseded by IL-2 receptor antagonists. The intensity of maintenance immunosuppression was reduced in cases of favourable HLA compatibility between donor–recipient pairs, as was the case for the patients in this study cohort.

### 2.2. Definition and Diagnoses

At the time of hospital admission for kidney transplantation, a diagnosis of BEN was established based on a combination of epidemiological, clinical, and laboratory criteria. Epidemiological criteria required rural residency for more than 15 years, while clinical criteria included a positive family history of chronic kidney disease (CKD) and/or UC, a personal history of UC, and the exclusion of other known or undetermined causes of ESKD. Laboratory data focused on the presence of severe anaemia disproportionate to the degree of renal impairment.

Following kidney transplantation and urological surgery, BEN diagnoses were reclassified according to consensus guidelines [11]. This reassessment was conducted in two stages: first, excluding post-transplant data, and subsequently incorporating post-transplant UTUC (Table S1, Supplementary Materials). As markers of AA exposure in tissue or blood were unavailable for routine diagnostics, AAN could not be definitively confirmed. Furthermore, histopathological findings of the native kidneys were non-informative, as all patients presented with advanced stage disease characterized by non-specific global fibrosis. Since all patients had ESKD, albumin-to-creatinine and α1-microglobulin-to-creatinine ratios were also deemed non-informative [11]. Of the 12 patients initially classified as having BEN, only two (one male and one female) resided in established endemic villages, and only one had a positive family history of CKD/UTUC.

A diagnosis of sporadic BEN was confirmed in UTUC patients post-surgery, following the exclusion of other nephropathies and undetermined causes of ESKD [9]. Due to advanced pathological changes in the native kidney tissue (extensive global fibrosis), histological confirmation of the primary diagnosis was not feasible.

UCs were staged and graded according to the World Health Organization (WHO) Classification of Tumours of the Urinary System and Male Genital Organs (5th edition, 2022) [13] and the TNM Classification of Malignant Tumours (8th edition) [14]. Carcinoma in situ (CIS) identified in association with high-grade UC was not analysed as a separate entity, as it represents a component of the same high-grade urothelial disease spectrum [15]. Histological analysis was performed on haematoxylin–eosin-stained sections.

### 2.3. Surgical Management of Urothelial Carcinoma

Patients with bladder carcinoma underwent transurethral electro-resection of the tumour. Patients diagnosed with UTUC were treated with open radical nephroureterectomy, comprising nephrectomy and ureterectomy with excision of the bladder cuff.

### 2.4. Outcomes

The primary outcome was defined as the detection of UC during post-transplant follow-up. Secondary outcomes included patient and graft survival, post-transplant complications, causes of death, and the concordance between the initial BEN diagnosis and the post-transplant reclassification. Recorded complications included acute rejection episodes, either clinically suspected or biopsy proven, managed with corticosteroid pulses, with additional plasma exchange and intravenous immunoglobulins administered in cases of antibody-mediated rejection. Other monitored outcomes included cardiovascular events, new-onset diabetes, infections requiring hospitalization, malignancies, and causes of graft failure. Baseline graft function, defined by stable serum creatinine and eGFR levels within the first post-transplant year, was assessed using the 2021 Chronic Kidney Disease Epidemiology Collaboration (CKD-EPI) equation to calculate the estimated glomerular filtration rate (eGFR) [16]. Patients underwent intensive urological surveillance, including urine cytology, ultrasonography of the urinary tract, computed tomography (CT) or magnetic resonance (MR) imaging of the abdomen and pelvis, and regular cystoscopy. Diagnostic approaches and surveillance protocols were further intensified in line with emerging evidence and the availability of advanced modalities, such as CT/MR urography and ureterorenoscopy over the last decade. Follow-up examinations were initially performed every three months and subsequently extended if no pathology was detected, in accordance with current clinical recommendations [11].

### 2.5. Statistical Analysis

Given the small sample size, all analyses were primarily descriptive and performed on available cases; missing data were not imputed. Continuous variables are presented as medians with interquartile ranges (IQRs), and categorical variables as frequencies and percentages. Time-to-event outcomes were summarized using Kaplan–Meier curves, with the date of transplantation defined as time zero for all survival analyses. For overall patient survival, death from any cause was defined as the event, while patients alive at the last follow-up or lost to follow-up were censored at the date of last clinical contact. For UC-free survival, the event was defined as the first histologically confirmed diagnosis of UC post-transplantation. In the UC-free survival analysis, death without a prior diagnosis of UC was treated as censoring; consequently, these Kaplan–Meier estimates are purely descriptive, as competing risks were not modeled. No formal hypothesis testing or multivariable modelling was performed due to limited statistical power. Statistical analyses were conducted using TIBCO Statistica, version 14.1.0.8 (TIBCO Software Inc., Palo Alto, CA, USA).

## 3. Results

### 3.1. Patient Characteristics, Epidemiological and Clinical Data

Patients’ epidemiological and clinical characteristics at the time of kidney transplantation are presented in Table 1 and Table S2A,B. Nine patients (75%) were male. All recipients had undergone chronic dialysis prior to transplantation (nine haemodialysis and three peritoneal dialysis), with a median dialysis duration of 16.5 months (IQR 9.8–48.4). The median age at transplantation was 58 years (IQR 47–66). Regarding smoking, one patient was a current smoker, four were former smokers, and seven were non-smokers. Pre-transplant comorbidities included hypertension in eight out of twelve patients, and diabetes in one; Notably, none of the patients had a history of myocardial infarction or stroke. The median duration of residence in rural farming regions was 24 years (IQR 23–34). Prior to admission for kidney transplantation, six of the twelve patients had been diagnosed with BEN (Table 1). Subsequent examination of native kidneys following nephroureterectomy revealed changes consistent with ESKD, although no specific histopathological features indicative of BEN were identified.

### 3.2. Reclassification of the BEN Diagnosis Post-Transplant According to the Consensus Statement

Table 1 summarizes patient characteristics, initial diagnoses at the time of kidney transplantation, and the reclassification of BEN diagnoses according to the consensus statement. Within the study cohort, only two patients (one male and one female) had resided in recognized BEN-endemic villages for more than 20 years (Figure S1). Seven patients reported a positive family history of CKD and/or UTUC, while two had a history of UTUC prior to transplantation. Patient 3 was reclassified as suspected BEN based on long-term residence in an endemic village (>20 years), the absence of indicators for other known causes of ESKD, severe anaemia, and normotension. Patient 10, also reclassified as having BEN, had long-term residence in a Croatian endemic village, a positive family history of CKD, and developed post-transplant UTUC, ultimately succumbing to metastatic UC 2.6 years after transplantation. Patient 12 presented with diabetic nephropathy and developed bladder cancer post-transplantation, without evidence of UTUC. Incorporating post-transplant follow-up data, patients 2, 4, 6, 8, and 9 were ultimately classified as having sporadic BEN (Table 1).

### 3.3. Transplant-Related Data and Post-Transplant Course

Except for one patient who underwent re-transplantation, all individuals in the study cohort received their first deceased-donor kidney allograft. Transplant-related characteristics and immunosuppressive regimens are detailed in Table S3. Six recipients (50%) had no HLA-DR mismatches, while five (41.7%) had a single HLA-DR mismatch; data were unavailable for one patient who underwent kidney transplantation in 1985, as HLA-DR typing was not routinely performed at that time. Panel-reactive antibody (PRA) data, assessed via complement-dependent cytotoxicity assays, were available for all but two patients from the 1980s; two patients exhibited PRAs (14% and 20%, respectively). Immunosuppressive therapy consisted of triple- or quadruple-drug regimens, including induction therapy in nine patients (75%), reflecting contemporary clinical practice. The median post-transplant follow-up was 6.8 years (range, 2.1–15.8 years). Two patients were lost to follow-up due to emigration after 6.0 and 6.6 years, respectively (Table 2). Acute rejection occurred in two patients (on postoperative days 7 and 13) and was successfully managed with corticosteroid pulse therapy. One recipient (Patient 7) experienced biopsy-proven antibody-mediated rejection and acute tubular necrosis on post-transplant day 4 (retransplant), requiring plasma exchange and intravenous immunoglobulins. No major cardiovascular events were recorded, except for one stroke in a recent transplant recipient re-classified as non-BEN. Infectious complications requiring hospitalization were frequent, primarily consisting of urinary tract and respiratory infections.

**Figure 2:**
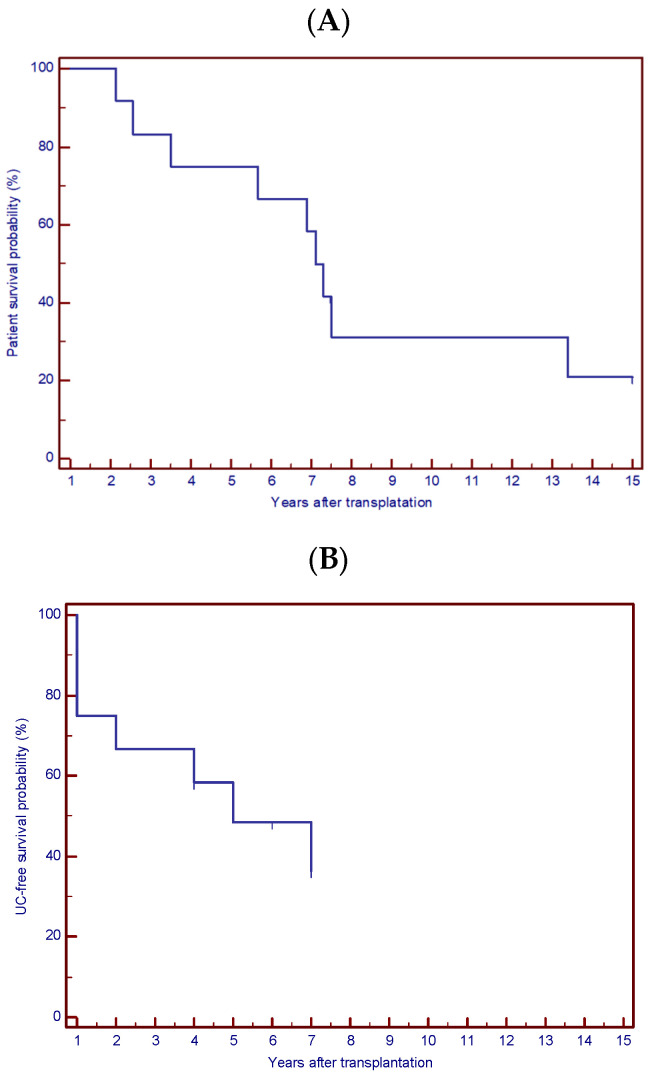
Cumulative patient survival probability (**A**), and urothelial carcinoma-free survival probability (**B**), in the study cohort after kidney transplantation at the Rijeka transplant center in Croatia from 1 January 1985 until 31 December 2024 (*n* = 12).

Throughout the follow-up period, all kidney allografts maintained stable function with no cases of graft loss. The cumulative patient survival probabilities at two and five years post-transplantation were 100% and 71%, respectively (Figure 1). Median patient survival was 7.1 years, while median UC-free survival was 5.0 years. Overall UC-free survival rates at 1, 2 and 5 years were 83.3%, 75.0%, and 41.7%, respectively. For the patient survival analysis (A), death from any cause was considered the event. Patients who were alive at the end of follow-up and those lost to follow-up were censored at the date of their last clinical contact. For UC–free survival analysis (B), the first diagnosis of UC after transplantation was considered the event (Figure 2). We emphasize that these analyses are descriptive only and should not be interpreted as robust survival estimates. Post-transplant complications, intervals to UC diagnosis, and causes of death are summarized in Table 2 and Table 3.

### 3.4. Characteristics of Urothelial Carcinoma and Treatment

UC developed in two patients prior to transplantation and in seven of the twelve recipients (58.3%) during the post-transplant follow-up; UC was the cause of death in four patients (33.3%). The disease involved the upper urinary tract, the bladder, or both, frequently demonstrating a multifocal and recurrent clinical course. The primary site of the first post-transplant UC was the bladder in four patients and the upper urinary tract in two. Patients with bladder carcinoma underwent transurethral electro-resection of the tumour, though none received adjuvant intravesical Bacillus Calmette–Guérin (BCG) therapy. Those diagnosed with UTUC were treated with radical nephroureterectomy, including nephrectomy and ureterectomy with excision of a bladder cuff. In Patient 2, UTUC developed 11.6 years before transplantation and recurred 7.6 years prior to the procedure. Treatment consisted of unilateral open radical nephroureterectomy followed by contralateral nephron-sparing surgery; however, the patient died 14.6 years after the second surgery due to metastatic disease. Patient 9 was treated for UC of the left renal pelvis and ureter (pT3N1, high grade) 16.6 years pre-transplant via open radical nephroureterectomy. He received adjuvant radiotherapy and remained recurrence-free. Following radical nephroureterectomy, two patients with metastatic disease received systemic chemotherapy. Immunosuppressive therapy was reduced in all affected patients. All nephroureterectomies involved the native urinary tract, except in Patient 6, who required partial resection of the renal allograft and ipsilateral nephroureterectomy due to UTUC involving the upper calyces of the transplanted kidney. In patients who underwent nephroureterectomy, the UC was typically multifocal. Both UTUC and bladder carcinomas were predominantly high-grade. In this cohort, multifocal UTUC was observed in 8 of the 10 removed renal units among the six patients with UTUC. Contralateral UTUC was detected in four of these six patients. Among the five patients with bladder carcinoma, four experienced recurrences. The clinical, pathological, and temporal characteristics of UC are summarized in Table 3.

## 4. Discussion

The present study contributes to the limited body of evidence regarding long-term kidney transplantation outcomes in patients with AAN/BEN, providing clinically relevant insights into diagnostic challenges, UC risk, and post-transplant prognosis. Rather than a statistical sample, this study represents a complete retrospective cohort of all eligible kidney transplant recipients at our center who originated from rural Balkan farming areas and were referred with a diagnosis of either BEN or CKD of unknown etiology. The inclusion period spans four decades, a timeframe during which significant advancements occurred in transplant eligibility criteria, immunosuppressive regimens, oncological surveillance strategies, and diagnostic approaches to BEN. While this longitudinal perspective is a strength, such temporal heterogeneity introduces potential confounding factors that must be considered when interpreting the long-term clinical outcomes.

### 4.1. Diagnostic Accuracy of BEN

Our results underscore the substantial difficulty in accurately diagnosing BEN in patients from rural farming areas presenting with ESKD of unknown origin, particularly when pathological and laboratory findings are inconclusive and molecular biomarkers, such as aristolactam-DNA adducts and the signature p53 mutation, are unavailable. In clinical practice, BEN is frequently diagnosed solely based on residence in an endemic village, often without consideration of the duration of exposure. Conversely, BEN is rarely considered in patients from non-endemic areas. Following a *post hoc* re-evaluation using consensus criteria and incorporating post-transplant UTUC development, we identified sporadic BEN in four patients not previously considered BEN cases. A significant challenge remains the limited physician familiarity with the precise geographic boundaries of endemic regions (Figure 1). Previously, the detection of aristolactam-DNA adducts, biomarkers of environmental exposure to AA, confirmed sporadic BEN in 11 UTUC patients originating outside established endemic villages [10]. In our cohort, six patients were diagnosed with BEN prior to transplantation. However, during post-transplant follow-up, only one could be reclassified as having BEN, while four were reclassified into sporadic or suspected categories. One patient lacked sufficient epidemiological or clinical data to support any BEN-related diagnosis. Misdiagnosing BEN can have serious clinical consequences, especially for kidney transplant candidates, as current consensus recommendations advocate for prophylactic bilateral nephroureterectomy in confirmed cases. In our cohort, six patients never developed UTUC. Conversely, two patients initially labelled as BEN who did not undergo prophylactic surgery subsequently developed post-transplant UTUC and died from metastatic disease. Interestingly, in one patient BEN was overlooked at admission despite a positive family history of both CKD and UTUC, and a prior history of UTUC surgery. His residence in a non-endemic region had precluded BEN as a differential diagnosis; we subsequently reclassified him as having sporadic BEN.

Admittedly, while the primary aim of this study was the reassessment of BEN, definitive confirmation of AA exposure was not available, as AA–DNA testing was not performed and histopathological evaluation was limited by advanced fibrosis. Furthermore, stored tissue samples were unavailable for retrospective molecular analysis. Establishing a diagnosis of sporadic BEN versus other forms of chronic tubulointerstitial nephropathy in the absence of molecular markers can be challenging; however, according to the international consensus document, such molecular confirmation is not considered obligatory for diagnosis. In this study, we applied the diagnostic criteria recommended by the expert group.

### 4.2. UTUC in AAN/BEN Patients

UTUCs are rare in the general population, with an estimated annual incidence in Western countries of almost two cases per 100,000 inhabitants, accounting for only 5–10% of all UCs. Notably, near half of incident cases in recent years have been muscle-invasive at diagnosis [17]. However, the prevalence of UTUC is not uniform. Exposure to AA has been associated with a six-fold increased risk of primary urinary tract malignancies (including both UC and renal cell carcinoma) and a 1.8-fold increased risk of recurrence [18]. In BEN regions, the incidence of UTUC is more than 100-fold higher than in non-endemic areas, confirming AA as a potent causative agent for both chronic tubulointerstitial nephropathy and UTUC [19,20]. In AAN (formerly referred to as Chinese herb nephropathy), UTUC has been reported in more than 40% of patients. These tumours are typically high-grade, frequently bilateral, and often multifocal [4,21]. While a female predominance observed in Belgian cohorts has been attributed to exclusive exposure through slimming regimens, data from Taiwan suggest a possible intrinsic female susceptibility [22,23,24,25]. In contrast, both genders appear equally affected in sporadic BEN [9,26]. In our cohort, UTUCs diagnosed prior to transplantation were high-grade and multifocal, with one patient developing contralateral disease. These findings align with previous reports indicating that while tumour stage and grade may not significantly differ between AAN and non-AAN UTUC, AA-associated carcinomas exhibit a markedly higher frequency of multifocality and recurrence. Similarly, Shan et al. found no significant differences in age, tumour location, surgical approach, pathologic grade, or stage between AAN and non-AAN patients [27].

### 4.3. Kidney Transplantation and UTUC in AAN/BEN Patients

Following kidney transplantation, the incidence of *de novo* malignancies ranges from 13% to 20%, representing an approximately 14-fold increased risk compared with the general population [28,29,30]. In AA-associated disease, UTUCs are frequently high-grade, multifocal, and diagnosed at advanced stages, with elevated rates of bilateral involvement and concomitant bladder cancer [18,27]. In contrast, synchronous bilateral UTUC is rare in non-AA-exposed patients (less than 20%), with concomitant bladder cancer reported in 20% to 30% of patients. AA exposure is specifically associated with multifocality of high-grade tumours (63.2%), a higher incidence of contralateral recurrence, and a greater frequency of *de novo* bladder cancers (39.5%) [18,31]. In our cohort, the observed UTUC incidence of 58.3% is striking, although this figure is derived from a limited number of cases. Given the restricted statistical power, definitive conclusions regarding the absolute magnitude of cancer risk in transplant recipients from rural farming areas remain elusive. Furthermore, intensified surveillance protocols during the last decade may have contributed to higher detection rates, not only in patients coming from rural farming areas but also in patients living in urban areas. Notably, multifocal high-grade UTUC was observed in all six patients who developed the disease, with four of them presenting with contralateral UTUC.

Four of the five patients with bladder UC (80%) experienced recurrences. Notably, one patient developed UTUC within the renal allograft, a rare phenomenon previously reported in BEN/AAN [32]. The median time from transplantation to UTUC diagnosis in our cohort was 48 months; although shorter than intervals reported in some BEN/AAN series, this remains consistent with the wide variability described in the literature [33,34,35,36]. Lemy et al. previously emphasized that patients remain at high risk for UTUC and bladder cancer even 15 years after the cessation of AA exposure [37]. Our findings support this, as UTUC was diagnosed an average of 17.6 years after patients left their respective farming areas, an interval likely reflecting the end of environmental AA exposure. These results support the fact that carcinogenic risk persists long after exposure has ceased. Furthermore, reports suggest that smoking may be less significant than AA exposure in the development of *de novo* UTUC after kidney transplantation in AAN [37,38]. Our observations in BEN patients align with these reports; among those who developed post-transplant UTUC, none were active smokers at the time of transplantation, two were former smokers, and four had never smoked. Surveillance protocols for UC were intensified throughout the observation period. In recent years, intravenous urography has been entirely superseded by CT and MR urography, which offer significantly higher diagnostic accuracy. Additionally, the development of flexible endoscopy, including ureterorenoscopy, has facilitated the detection of tumours within the renal pelvis and calyces. Depending on their size, small lesions can now be managed endoscopically as part of a nephron-sparing (kidney-sparing) surgical approach.

### 4.4. Post-Transplant Clinical Course and Outcomes

The rates of acute rejection and infectious episodes in our cohort were comparable to those reported in previous studies [39]. Cardiovascular morbidity remained low, aligning with earlier observations suggesting a reduced cardiovascular risk profile in patients with BEN [40]. Despite favourable graft outcomes, mortality was high among patients who developed UC. While immunosuppression was consistently reduced in these cases, it remains unclear whether regimens containing mTOR inhibitors influenced oncological outcomes [41]. Intravesical BCG therapy for non-muscle invasive bladder cancer was avoided to prevent potential systemic BCG infection, a concern also noted in patients with AAN [37]. Our findings are consistent with an earlier study of BEN transplant recipients, which demonstrated that patients who did not undergo prophylactic nephroureterectomy frequently developed post-transplant UTUC, identifying it as a leading cause of death [32]. Conversely, research on AAN patients who underwent preventive bilateral nephroureterectomy has shown excellent long-term patient and graft survival rates [37,39]. Furthermore, Zhang et al. reported that simultaneous bilateral radical nephroureterectomy was associated with improved survival compared to unilateral surgery [35].

### 4.5. Strengths and Limitations

The principal strength of this study is the extensive follow-up period, which allowed for the evaluation of late oncological outcomes following kidney transplantation. Furthermore, the comprehensive *post hoc* reclassification of BEN diagnoses using consensus criteria highlights real-world diagnostic challenges with direct clinical relevance. This manuscript provides valuable observational data regarding the diagnostic complexity of BEN and the occurrence of UC post-transplantation. However, several limitations must be acknowledged. The small sample size, inherent diagnostic uncertainty, and the heterogeneity of the cohort substantially limit the strength of the conclusions. The study included only 12 patients collected over four decades, with only one case of confirmed BEN prior to transplantation. Consequently, the results should be interpreted as exploratory observations rather than definitive evidence supporting broader clinical recommendations. Additionally, the absence of molecular biomarkers for AA exposure precluded definitive diagnostic confirmation. Nevertheless, given the rarity of BEN and the extended observation period, these findings offer meaningful insights into the long-term clinical management of these patients.

## 5. Conclusions

This long-term, single-centre study demonstrates the frequent misclassification of BEN among kidney transplant candidates from rural Balkan regions. The application of contemporary consensus criteria reclassified BEN in only a minority of cases initially labelled as such, while other patients remained underdiagnosed. Despite excellent long-term graft survival, aggressive and multifocal UC was common and represented the leading cause of death in transplant recipients. These findings indicate that AA-related carcinogenic risk persists for decades after exposure and may manifest outside traditionally recognized endemic areas. Consequently, increased clinical awareness of sporadic BEN is essential. Lifelong, intensive surveillance should be mandatory for confirmed BEN cases, as well as for patients from other rural Balkan farming regions at risk for sporadic BEN.

### 5.1. Evidence Confirmed

This study confirms that kidney transplant recipients within the BEN/AAN spectrum remain at high risk for aggressive, multifocal, synchronous, and metachronous UC long after transplantation and the presumed cessation of AA exposure. This carcinogenic risk persists for decades and represents the primary cause of mortality in this population, despite excellent long-term kidney allograft survival.

### 5.2. Added Value of the Study

By systematically reclassifying BEN diagnoses using contemporary consensus criteria, this study provides real-world evidence of the clinical consequences associated with diagnostic misclassification. It highlights sporadic BEN as an underrecognized but clinically relevant entity occurring outside traditional endemic regions. Furthermore, it offers unique long-term follow-up data that link diagnostic reassessment with specific transplant-related and oncological outcomes.

### 5.3. Implications of Our New Evidence

Patients with ESKD stage who have resided in rural farming villages for more than 20 years should undergo meticulous evaluation to confirm or exclude BEN/AAN. Prophylactic bilateral nephroureterectomy should be strongly considered in patients with a confirmed BEN. Post-transplantation, patients from all Balkan rural farming regions, not only those from established endemic areas, require lifelong, intensive urological surveillance to ensure the prompt identification of UTUC. Additionally, as bladder cancer may develop decades after the cessation of AA exposure, it must remain a key focus of long-term clinical monitoring.

### 5.4. Bullet Points

Balkan nephropathy is frequently misclassified in kidney transplant candidates; applying strict consensus criteria reveals significant over- and underdiagnosis, including sporadic cases outside traditionally recognized regions.

Despite excellent long-term graft survival, aggressive and multifocal urothelial carcinoma remains a common complication and the leading cause of mortality in this patient population.

Carcinogenic risk persists for decades after the cessation of AA exposure, underscoring the necessity of prophylactic surgery in confirmed cases and lifelong, intensive urological surveillance.

## Figures and Tables

**Table 1:** Patients’ characteristics and reclassification of BEN diagnosis according to the consensus statement in the study cohort who underwent kidney transplantation at the Rijeka transplant center in Croatia from 1 January 1985 until 31 December 2024 (*n* = 12) [11].

Pt	BEN Diagnoses Before Admission for Tx	Living in Rural Area >20 y	BEN Village	Family History CKD/UTUC	Other CKD	UTUC Pre-Tx	Anaemia/Pre-Tx Blood Transfusion	Normal Blood Pressure	Smoking Status	Re-Classified with Post-Tx Follow-Up Data
1	BEN	Yes	No	No/No	No	No	Yes/Yes	No	Yes	Non-BEN
2	No	Yes	No	No/No	No	Yes	Yes/Yes	Yes	No	Sporadic
3	BEN	Yes	Yes	No/No	No	No	Yes/Yes	Yes	No	Suspected
4	No	Yes	No	Yes/No	No	No	Yes/Yes	yes	No	Sporadic
5	No	Yes	No	No/No	No	No	Yes/No	Yes	No	Non-BEN
6	BEN	Yes	No	Yes/No	No	No	Yes/Yes	No	No	Sporadic
7	No	Yes	No	No/No	No	No	Yes/Yes	No	No	Non-BEN
8	No	Yes	No	Yes/Yes	No	No	Yes/Yes	No	Ex	Sporadic
9	No	Yes	No	Yes/Yes	No	Yes	Yes/Yes	No	Ex	Sporadic
10	BEN	Yes	Yes	Yes/No	No	No	Yes/No	No	No	BEN
11	BEN	Yes	No	Yes/No	No	No	Yes/No	No	Ex	Non-BEN
12	BEN	Yes	No	Yes/No	Yes	No	Yes/Yes	No	Ex	Non-BEN

Pt, patient; y, years; CKD, chronic kidney disease; UTUC, upper urinary tract cancer; Tx, transplantation.

**Table 2:** Post-transplant complications and outcomes in patients of the study cohort after kidney transplantation at the Rijeka transplant center in Croatia from 1 January 1985 until 31 December 2024 (*n* = 12).

Pt	Year of Tx	Acute Rejection	New-Onset HTN	New-Onset DM	MI	Stroke	Infections *	Follow-Up (Years)	Outcome	Cause of Death
1	1985	Yes	No	No	No	No	No	6.6 **	Lost	-
2	1986	Yes	No	No	No	No	Respiratory	7.1	Deceased	Urothelial carcinoma
3	1986	No	No	No	No	No	No	6.0 **	Lost	-
4	1994	No	No	No	No	No	No	5.7	Deceased	Urothelial carcinoma
5	1998	No	No	No	No	No	Respiratory	6.9	Deceased	Sudden death
6	1998	No	No	Yes	No	No	Urinary	15.8	Deceased	Plasmacytoma
7	2006	Yes	No	No	No	No	Urinary	2.1	Deceased	Urothelial carcinoma
8	2011	No	No	No	No	No	Urinary, respiratory	14.8	Alive	–
9	2013	No	No	No	No	No	No	3.5	Deceased	Colonic perforation
10	2013	No	No	No	No	No	Urinary	2.6	Deceased	Urothelial carcinoma
11	2014	No	No	No	No	No	Respiratory, urinary	7.3	Deceased	COVID-19
12	2018	No	No	No	No	Yes	No	7.4	Alive	–

Pt, patient; HTN, hypertension; DM, diabetes mellitus; MI, myocardial infarction; * Infections listed include only those requiring hospitalization. ** follow-up was discontinued due to patient emigration.

**Table 3:** Characteristics of urothelial carcinoma in patients of the study cohort who received a kidney transplant at the Rijeka transplant center in Croatia from 1 January 1985 until 31 December 2024 (*n* = 12).

Patient	UC Diagnosis Post-Tx (Years)	UC Location		TNM Stage	Grade	Metastatic UC Post-Tx (Years)
2	7.0	Right pelvis *				7.0
4	4.8	Left ureter **		NA	HG	5.2
6	1.1		Bladder	pT1	HG	
	2.9	Left ureter **		pT1	HG	
	6.6	Allograft pelvis ***		pT1	LG	
7	0.4		Bladder	pT1	HG	0.9
8	4.4	Right pelvis and ureter **		pT3	HG	
	5.6		Bladder	pT1	HG	
	9.9	Left pelvis and ureter **		pT2	HG	
10	0.2		Bladder	pT1	HG	2.5
	0.9	Left pelvis and ureter **		pT2	HG	
	1.5	Right ureter **		pT1	LG	
12	2.1		Bladder	pT1	NA	

UC, urothelial carcinoma; Tx, transplantation; TNM, tumour nodes metastasis; HG, high-grade; LG, low-grade; NA, not available. * 11.6 years pre-Tx high-grade UC of the left pelvis and ureter, open radical nephroureterectomy with excision of the bladder cuff; 7.6 years pre-Tx high-grade UC of the right pelvis, partial kidney resection. ** open radical nephroureterectomy with excision of the bladder cuff. *** partial resection of the allograft and ipsilateral radical nephroureterectomy.

## Data Availability

The original contributions presented in this study are included in the article/Supplementary Materials. Further inquiries can be directed to the corresponding authors. Data on all kidney transplantations at the Reference Center in Croatia have been reported to national databases, since 1985 to the Collaborative Transplant Study (CTS, www.ctstransplant.org), and since 2007 to Eurotransplant.

## Supplementary Materials

The following supporting information can be downloaded at: https://www.mdpi.com/article/10.3390/jcm15072558/s1, Table S1: Classification of Balkan nephropathy [11]; Table S2A: Epidemiological and baseline clinical characteristics at the time of kidney transplantation; Table S2B: Epidemiological and baseline clinical characteristics at the time of kidney transplantation; Table S3: Transplant-related characteristics and immunosuppressive therapy.

## Abbreviations

The following abbreviations are used in this manuscript:

AA	Aristolochic acid
AAN	Aristolochic acid nephropathy
BEN	Balkan nephropathy
CIS	Carcinoma in situ
CKD	Chronic kidney disease
ESKD	End-stage kidney disease
PRA	Panel-reactive antibodies
UC	Urothelial carcinoma
UTUC	Upper urinary tract urothelial carcinoma
Tx	Transplantation
