# Supplementary material for: Long-Term Outcome of Kidney Transplant Patients from Rural Farming Areas with Balkan Nephropathy—A Single-Centre Report
Source: J Clin Med. 2026 Mar 27;15(7):2558. doi: 10.3390/jcm15072558 (PMC13073947; doi:10.3390/jcm15072558)
Supplement: Supplementary file 1 [file jcm-15-02558-s001.zip › jcm-4190271-supplementary.pdf]

Supplementary materials

Table S1. Classification of Balkan nephropathy [11]

**I. Diseased/affected**<br>1. Biopsy proven/indicative of BEN<br>or<br>2. Residency in a BEN household >20 years<br>+ tubular proteinuria[1]<br>+ decreased eGFR<br>+ anemia[2]<br>or<br>3. Residency in BEN village >20 years<br>+ UUC<br>+ tubular proteinuria[1]	**II Suspected BEN**<br>1.Residency in BEN household >20 years<br>+ reduced eGFR<br>+ anemia[2]<br>or<br>2. Residency in BEN household >20 years<br>+ tubular proteinuria[1]<br>or<br>3. Residency in BEN village >20 years<br>+ UUC
**III. High risk group for EN**<br>1.Residency in EN households >20 years<br>2. Residency in households with sporadic/suspected EN cases >20 years	**IV. Sporadic EN[3]**<br>Biopsy proven/indicative of EN in patient with UUC outside of the endemic region or in member of their household

[1] α-1MCR >31.5 mg/g and α -1MCR/UAC=0.91.

[2] Haemoglobin <120 g/L for men and women >50 years, and <110 g/L for women ≥50 years.

[3] Subjects with chronic interstitial nephropathies where other causes should be excluded (reflux nephropathy, chronic pyelonephritis, recurrent pyelonephritis, hypertensive, nephrosclerosis, exposure to lead, cadmium, herbs containing AA, cyclosporin A, ifosfamide, pamidronate, lithium and nitrosoureas, heavy use of NSAID).

Table S2A. Epidemiological and baseline clinical characteristics in patients of the study cohort at the time of kidney transplantation at the Rijeka transplant centre in Croatia from 1 January 1985 until 31 December 2024 (n=12)

Pt	Age (years)	Village of residence	Country	Years in farming area	Smoking Status	PreTx HTN	PreTx DM	PreTx Strok e	PreTx MI	Other CKD
1	29	Žabari	Serbia	19	Yes	Yes	No	No	No	No
2	66	Romanovci	BiH	46	No	No	No	No	No	No
3	61	Lužani	Croatia	24	No	No	No	No	No	No
4	36	Zym (Zjum)	Kosovo	24	No	No	No	No	No	No
5	64	Vukšić Donj	BiH	63	No	No	No	No	No	No
6	45	Otok	Croatia	38	No	Yes	No	No	No	No
7	49	Vraniq	Kosovo	30	No	Yes	No	No	No	No
8	55	Sinajë	Kosovo	23	Former	Yes	No	No	No	No
9	53	Gjakova	Kosovo	24	Former	Yes	No	No	No	No
10	76	Stupnički .Kuti	Croatia	22	No	Yes	No	No	No	No
11	68	Bečic	Croatia	23	Former	Yes	No	No	No	No
12	66	Miloševac	BiH	21	Former	Yes	Yes	No	No	Yes

Pt, patient; M, male; F, female; PreTx, pre-transplant; HTN, hypertension; DM, diabetes mellitus CVI, cerebrovascular incident; MI, myocardial infarction; CKD, chronic kidney disease; HD, haemodialysis; PD, peritoneal dialysis.

Table S2B. Epidemiological and baseline clinical characteristics in patients of the study cohort at the time of kidney transplantation at the Rijeka transplant centre in Croatia from 1 January 1985 until 31 December 2024 (n=12)

Pt	Gender	BMI (kg/m^2)	BP (mmHg)	Heart Rate (bpm)	Erythro-cytes ($\times 10^{12}/L$)	Haemoglob in (g/L)	Haemato-crit	Dialysis Modality	Dialysis vintage (months)
1	M	23.5	180/115	86	2.3	80	0.29	HD	48,3
2	M	24.2	130/80	88	2.6	82	0.26	HD	10,0
3	M	22.7	125/75	92	1.8	58	0.18	HD	10,3
4	M	24.2	200/120	80	2.5	85	0.25	HD	7,9
5	F	21.9	118/84	90	2.5	85	0.30	HD	48,5
6	M	21.9	190/100	90	3.0	89	0.26	HD	25,3
7	M	25.4	150/70	65	2.57	79	0.23	HD	213,7
8	F	22.4	150/80	88	4.36	127	0.4	HD	79,3
9	M	24.3	130/70	80	3.88	115	0.34	HD	21,6
10	F	25.2	140/80	74	3.15	114	0.35	PD	9,5
11	M	26.8	160/90	70	4.45	125	0.37	PD	10,4
12	M	26.6	187/103	89	3.78	107	0.3	PD	0,1

Pt, patient; BMI, body mass index; BP, blood pressure

Table S3. Transplant-related characteristics and immunosuppressive therapy in patients enrolled in this study who underwent kidney transplantation at the Rijeka transplant centre in Croatia from 1 January 1985 until 31 December 2024 (n=12)

Patient	Year of Tx	HLA-DR Mismatches	Total HLA-A, -B, -DR Mismatches	PRA (%)	Induction Therapy	Steroids	CNI	Antimetabolite	mTOR Inhibitor
1	1985	NA	NA	-	ALG	Yes	–	AZA	–
2	1986	1	3	0	ALG	Yes	–	AZA	–
3	1986	1	4	-	ALG	Yes	–	AZA	–
4	1994	0	1	0	None	Yes	CyA	AZA	–
5	1998	0	2	0	None	No	CyA	AZA	–
6	1998	1	3	0	None	Yes	CyA	AZA	–
7*	2006	1	2	14	IL2RA	Yes	Tac	MPA	–
8	2011	0	3	20	Thymoglobulin	Yes	Tac	MPA	–
9	2013	0	2	0	IL2RA	Yes	–	MPA	Sirolimus
10	2013	1	4	0	IL2RA	No	Tac	MPA	–
11	2014	0	3	0	IL2RA	Yes	Tac	–	Everolimus
12	2018	0	2	0	IL2RA	Yes	Tac	MPA	–

PRA, panel-reactive antibodies; ALG, antilymphocyte globulin; IL2RA, interleukin-2 receptor antagonist; CNI, calcineurin inhibitor; CyA, cyclosporine A; Tac, tacrolimus; AZA, a□athioprine; MPA, mycophenolate mofetil or mycophenolic acid; mTOR, mammalian target of rapamycin.

* The patient underwent a first kidney transplant in India at 35 years of age, which was complicated by transfusion-acquired hepatitis C and allograft rejection after one year, followed by a return to haemodialysis.
